# Anticoagulant Activity of Polyphenolic-Polysaccharides Isolated from *Melastoma malabathricum* L.

**DOI:** 10.1155/2014/614273

**Published:** 2014-05-20

**Authors:** Li Teng Khoo, Faridah Abas, Janna Ong Abdullah, Eusni Rahayu Mohd Tohit, Muhajir Hamid

**Affiliations:** ^1^Department of Microbiology, Faculty of Biotechnology and Biomolecular Sciences, Universiti Putra Malaysia, 43400 Serdang, Selangor, Malaysia; ^2^Department of Food Science, Faculty of Food Science and Technology, Universiti Putra Malaysia, 43400 Serdang, Selangor, Malaysia; ^3^Institute of Biosciences, Universiti Putra Malaysia, 43400 Serdang, Selangor, Malaysia; ^4^Department of Pathology, Faculty of Medicine and Health Sciences, Universiti Putra Malaysia, 43400 Serdang, Selangor, Malaysia

## Abstract

*Melastoma malabathricum* Linn. is a perennial traditional medicine plants that grows abundantly throughout Asian countries. In this study, *M. malabathricum* Linn. leaf hot water crude extract with anticoagulant activity was purified through solid phase extraction cartridge and examined for the bioactive chemical constituents on blood coagulation reaction. The SPE purified fractions were, respectively, designated as F1, F2, F3, and F4, and each was subjected to the activated partial thromboplastin time (APTT) anticoagulant assay. Active anticoagulant fractions (F1, F2, and F3) were subjected to chemical characterisation evaluation. Besides, neutral sugar for carbohydrate part was also examined. F1, F2, and F3 were found to significantly prolong the anticoagulant activities in the following order, F1 > F2 > F3, in a dose dependent manner. In addition, carbohydrate, hexuronic acid, and polyphenolic moiety were measured for the active anticoagulant fractions (F1, F2, and F3). The characterisation of chemical constituents revealed that all these three fractions contained acidic polysaccharides (rhamnogalacturonan, homogalacturonan, and rhamnose hexose-pectic type polysaccharide) and polyphenolics. Hence, it was concluded that the presence of high hexuronic acids and polysaccharides, as well as polyphenolics in traditional medicinal plant, *M. malabathricum*, played a role in prolonging blood clotting in the intrinsic pathway.

## 1. Introduction

*Melastoma malabathricum* Linn. is a small shrub belonging to the Melastomataceae family [1]. This evergreen plant grows wildly and abundantly in open fields, lowlands, and mountainous forests throughout the tropical and subtropical regions. In Malaysia,* M. malabathricum *Linn. is known as “senduduk,” and this species is widely accepted as a traditional medicinal plant [2].

The whole* M. malabathricum* Linn. plant has been reported to be used as a folklore medicine. Decoction from the leaves is the most favourable as it is believed to be able to treat diarrhoea and dysentery and heal wounds and other ailments [3]. Besides, scientific reports have shown that the leaf crude extract of* M. malabathricum* Linn. exhibited antidiarrheal [4] as well as antinociceptive, anti-inflammatory, and antipyretic activities [5, 6].

Manicam et al. [7] have shown that* M. malabathricum* Linn. hot water extract has an outstanding anticoagulant activity. To our best knowledge, however, there has been no report on the chemical constituents of* M. malabathricum *Linn.hot water extract, which exhibits anticoagulant activity. Previous literature has also proven that the anticoagulant activity of* Lythrum salicaria *and* Porana volubilis *is contributed by acidic polysaccharides and polyphenolic compounds [8, 9]. In this study, it was hypothesised that the fractions from hot water extract of* M. malabathricum *Linn. leaf contain similar chemical constituents which demonstrate similar anticoagulant activity that is probably better than other higher plants such as* L. salicaria *and* P. volubilis. *Thus, in continuum with the previous study, the hot water crude extract of* M. malabathricum* Linn. leaf was fractionated, and anticoagulant activities of fractionated fractions were evaluated. At the same time, carbohydrate, hexuronic acid, and polyphenolic moiety in the active anticoagulant fractions were also measured. Overall, this study revealed that the fractions of* M. malabathricum *Linn. contained high amounts of carbohydrate, hexuronic acid, and polyphenolic, which are responsible for the anticoagulant activity. Such a discovery is crucial information as it provides clues for potential treatment of blood coagulant disorders.

## 2. Material and Methods

### 2.1. Materials

Standard human normal pooled plasma, STA-PTT automated reagent, and 0.025 M calcium chloride were purchased from Diagnostica Stago (Asnieres-sur-Seine, France), while sodium bicarbonate, sodium borohydride, gallic acid, carbazole, glucuronic acid, arabinose, galactose, mannose, rhamnose, xylose, dextran standards, trifluoroacetic acid (TFA), and heparin sodium salt were bought from Sigma-Aldrich (Missouri, USA). Phenol, acetonitrile, ammonium hydroxide, glacial acetic acid, acetic anhydride, dimethyl sulfoxide (DMSO), and Folin-Ciocalteu's reagent were purchased from Merck (New Jersey, USA). Concentrated sulphuric acid and glucose were purchased from Fisher Scientific (Massachusetts, UK), and sodium borate de carbohydrate was purchased from Biobasic (Ontario, Canada).

### 2.2. Plant Material

Fresh matured* M. malabathricum *Linn. leaves were collected between September and October 2009 from Lebuh Silikon, Universiti Putra Malaysia, in Serdang, Selangor, Malaysia. The samples were identified by a botanist and a voucher specimen was deposited at the Herbarium Biodiversity Unit, Institute of Biosciences, Universiti Putra Malaysia, under the reference number SK 1717/09.

### 2.3. Extraction and Fractionation of the Plant Material

The hot water extraction from the leaf of* M. malabathricum *was performed according to the method described by Manicam et al. [7]. Briefly, fine pieces of* M. malabathricum *leaves (500 g) were refluxed for 5 h at 100°C with 1 L deionised water. After 5 h, the hot water extract was filtered using Whatman Grade number 1 filter paper (Whatman, US), concentrated into dryness, lyophilised, and stored at −20°C until further analysis. Next, lyophilised crude extract (0.5 g) was dissolved in 100% deionised water and loaded into 10 g of C_18_, Sep-Pak Cartridge (Water, Massachusetts, USA). Four different ratios of water to acetonitrile (95 : 5, 90 : 10, 80 : 20, and 50 : 50, v/v) were passed through the cartridge. The analysts of interests for each fraction were collected, dried and lyophilized. Four fractions designated as F1, F2, F3, and F4 were obtained. Each fraction was reconstituted with distilled water at different concentrations for anticoagulant activity assay and chemical characterisation.

### 2.4. Anticoagulant Assay

*In vitro *intrinsic anticoagulant pathway was measured by activated partial thromboplastin time (APTT). Standard human normal pooled plasma was spiked with equal volume of different concentrations of* M. malabathricum *crude extract and fractions (1 mg/mL, 500 *μ*g/mL, 250 *μ*g/Ml, and 125 *μ*g/mL). Meanwhile, STA-PTT automated reagent was reconstituted and subjected to APTT assay, together with 0.025 M calcium chloride. The APTT assay was analysed using STA Compact coagulation analyser (Diagnostica Stago, Asnieres-sur-Seine, France) according to the manufacturer's protocol. The anticoagulant activity was measured by using a parallel standard curve based on the standard calibration curve for heparin sodium salt (140 IU/mg).

### 2.5. Chemical Characterisation

Total carbohydrate content was determined by using a modified phenol-sulphuric acid assay [10]. Absorbance was measured on Mindray microplate reader (Shenzhen, China) and glucose was used as a standard. Total phenolic content was measured by using a modified Folin-Ciocalteu method [11]. The content of hexuronic acid was determined by carbazole assay [12]. Absorbances of total phenolic and hexuronic acid contents were measured on Novaspec II visible spectrophotometer (Nicosia, Greek). Gallic acid and glucuronic acid were used as standards, respectively. Infrared spectrum was recorded with Perkin-Elmer Spectrum 100 FT-IR infrared spectrometer (Massachusetts, USA) and 16 scans were recorded with 4 cm^−1^ resolution. The ^1^H NMR spectra were obtained from 500 MHz Varian INOVA NMR spectrometer (California, USA). All the ^1^H NMR samples were dissolved in deuterium oxide (D_2_O).

### 2.6. Monosaccharide Compositions Analysis

Neutral sugar compositions were identified and quantified through gas chromatography (GC) analysis. Polysaccharides were subjected to hydrolysation, reduction, and acetylation [13, 14]. Active anticoagulant samples (5 mg) were hydrolysed with 2 M TFA at 100°C for 2 h and dried. This was followed by reconstituting the samples with 200 *μ*L water and adding 20 *μ*L allose as an internal standard. Next, 20 *μ*L of 15 M ammonium hydroxide and 1 mL of 0.5 M sodium borohydride solution in dimethyl sulfoxide were added. The samples were then left overnight at 4°C after being incubated in 40°C water bath for 90 min. On the following day, the samples were prewarmed at 40°C for 5 min. Subsequently, 100 *μ*L of glacial acetic acid, 200 *μ*L of 1-methylimidazole, and 1 mL of acetic anhydride were added. The samples were incubated at 40°C for 10 min to allow completion of acetylation. Finally, 2.5 mL of water and 1 mL of dichloromethane were added and centrifuged at 1000 ×g for 10 min. The lower dichloromethane part was analysed using GC. Meanwhile, arabinose, glucose, galactose, mannose, rhamnose, and xylose were used as standards. Aditol hexa-acetates were analysed using Agilent 6890 gas chromatography (Santa Clara, USA) with BPX-70 column (30 m × 0.32 mm I.D., 0.25 *μ*m film thicknesses, SGE, Australia). Helium was used as gas carrier on column injection and flame ionisation detector was utilized as a detector. The temperature was programmed at 50°C and maintained for 30 s, increased to 170°C at 50°C/min and finally to 230°C at 2°C/min before it was maintained for 5 min. The inlet temperature was 250°C, with a flow rate of 1 mL/min.

### 2.7. Molecular Mass Determination

Molecular mass of active anticoagulant samples was determined through Jasco high performance liquid chromatography (HPLC LC-1500 series) with refractive index detector (Maryland, USA). Phenomenex polysep-GFC-P-4000 (California, USA) was used with the flow rate at 0.8 mL/min, while the mobile phase was deionised water. Dextran standards of various molecular masses from 5 kDa to 270 kDa were used as standards for calibration.

### 2.8. Statistical Analysis

Data are shown as mean ± S.E.M. (*n* = 3) and analysis was performed using one-way analysis of variance (ANOVA), followed by* post hoc* Dunnett's multiple range tests to determine the means of significance from the control (Prism 5.0, GraphPad Software Inc., California, USA). Significant differences for all the data sets were measured and designated as *P < 0.05, **P < 0.01, and ***P < 0.001.

## 3. Results and Discussion

A previous study by Manicam et al. [7] showed that* M. malabathricum *Linn. leaf hot water extract exhibited an excellent anticoagulant activity. Therefore, in this study, the hot water extract of* M. malabathricum* Linn. leaf was fractionated, while the anticoagulant activity of each fraction was evaluated. Chemical compositions of active anticoagulant fractions were also elucidated.

**Figure 1:**
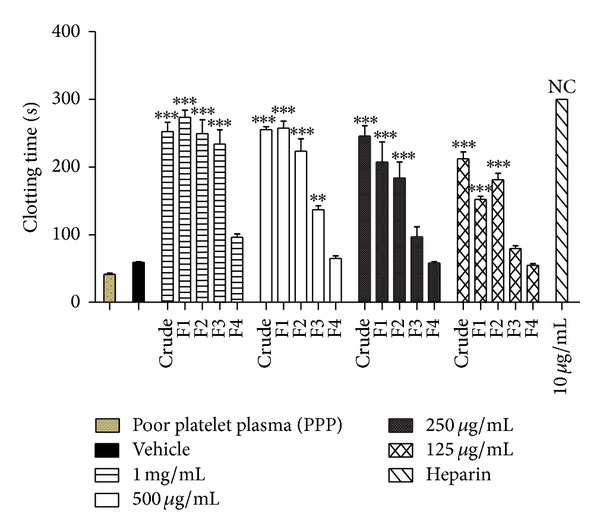
Activated partial thromboplastin time (APTT) of* Melastoma malabathricum* Linn. leaf extract purified via solid phase extraction (SPE) into F1, F2, F3, and F4. Crude extract and fractions (1 mg/mL, 500 *μ*g/mL, 250 *μ*g/mL, and 125 *μ*g/mL) were tested on human normal pooled plasma. Heparin sodium salt from porcine intestinal mucosa (140 USP units mg^−1^) was used as a positive control. Data represent means ± S.E.M. of three independent experiments. Significant differences compared to the control group (normal pooled plasma with deionised water, vehicle) are designated as **P < 0.05 and ***P < 0.001. NC: no coagulation.

The dark brown material (lyophilised crude extract) was fractionated into four fractions with increasing solvent polarity. The fractions were labelled as F1, F2, F3, and F4. The anticoagulant activity of each fraction was determined by automated partial thromboplastin time (APTT) assay. This is in continuum with Manicam et al.'s report, which showed that* M. malabathricum *Linn. leaf hot water extract possessed outstanding prolongation blood clotting time verified by the APTT assay. APTT is able to measure the inhibition of intrinsic factors of blood coagulation pathway such as F XII, XI, IX, VIII, and V [15]. Overall, all the fractions showed significant prolonged intrinsic blood clotting time, except for F4 (Figure 1). Hence, F4 was not considered for further chemical characteristics evaluation. Meanwhile, F1 was shown to be the most active anticoagulant fraction as it significantly prolonged APTT at a concentration as low as 125 *μ*g/mL. It is noteworthy to highlight that F1 was more active compared to the crude extract. Specifically, the clotting time for F1 was about 273.33 ± 3.28 s (*P* < 0.001) and 264.15 ± 2.34 s (*P* < 0.001) compared to the crude extract, which exhibited 252.51 ± 2.01 s (*P* < 0.001) and 255.56 ± 3.68 s (*P* < 0.001) at 500 *μ*g/mL and 1 mg/mL, respectively. Similarly, F2 also showed a similar anticoagulant characteristic as F1. It significantly prolonged the blood clotting time at a concentration as low as 125 *μ*g/mL. The prolonged clotting time at 1 mg/mL for F2 was 249.37 ± 3.45 s (*P* < 0.001). However, F3 was only able to significantly inhibit blood clotting at 1 mg/mL and 500 *μ*g/mL, with 234.14 ± 0.99 s (*P* < 0.001) and 137.11 ± 0.58 s (*P* < 0.05), respectively.

**Figure 2:**
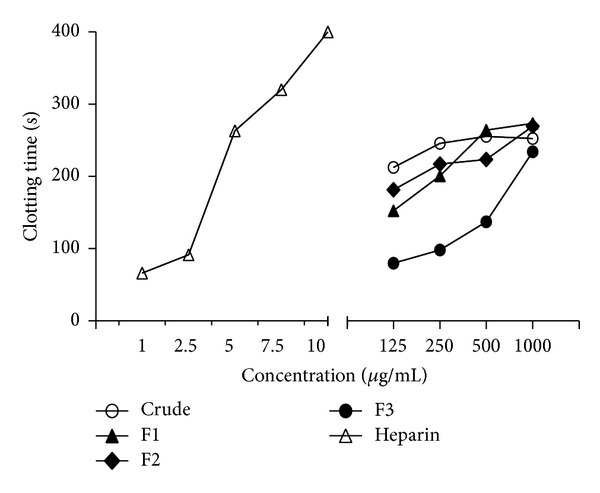
Anticoagulant activities of* Melastoma malabathricum *Linn. leaf crude extract, F1, F2, and F3, in comparison with the activity of heparin sodium salt (140 IU/mg), measured by activated partial thromboplastin time (APTT). F1, F2, and F3 were purified by solid phase extraction (SPE) from* M. malabathricum *Linn. leaf hot water crude extract. Data represent means of three independent experiments.

The anticoagulant activity for all the fractions was subsequently measured using the standard calibration curve of unfractionated heparin (140 IU/mg). As summarised in Figure 2, the anticoagulant activities for F1, F2, and F3 were 0.90 IU/mg, 0.89 IU/mg, and 0.77 IU/mg, respectively. The two fractions exhibited more active anticoagulant profiles compared to* Lythrum salicaria *(0.17 IU/mg) and* Filipendula ulmaria *(0.5 IU/mg) and were therefore reported as anticoagulant medicinal higher plants [16].

The chemical characterisation was carried out to have a better understanding of the chemical compositions for active anticoagulant fractions (F1, F2, and F3). F1 was a dark brown gum, while F2 was a brown material, and F3 was a light brown material. The yields for F1, F2, and F3 were 54.17%, 14.97%, and 8.8% (w/w) of dry crude extract, respectively.

Generally, carbohydrates, phenolic acids, and uronic acids were presented and they served as the main constituents for F1, F2, and F3 (Table 1). It is interesting to note that all active anticoagulant fractions were rich in carbohydrate contents. The* M. malabathricum *Linn. hot water extract which was fractionated with increasing solvent polarity had decreased carbohydrate compositions in the F1, F2, and F3. From the results, F1 with the most potent anticoagulant fraction yielded the highest compositions of carbohydrate and uronic acid contents. The occurrences of carbohydrate and uronic acid compositions of fractions were correlated with the anticoagulant activities. The anticoagulant activities of the fractions decreased as the carbohydrate and uronic acid contents decreased in the following sequence F1, F2, and F3.

On the other hand, F3 with moderate amounts of carbohydrate and uronic acid contents compared to F1 and F2 also displayed significant anticoagulant activity. This might be due to the presence of high percentage of phenolic acids in F3. Phenolic acids together with carbohydrates and uronic acids had been suggested to play a role in anticoagulant activities by Pawlaczyk et al. [17, 18] and Mozzicafreddo et al. [19].

Besides, three types of neutral sugars were being identified from F1, F2, and F3 active anticoagulant fractions. These findings suggest that rhamnogalacturonan, homogalacturonan, and rhamnose hexose types of pectic polysaccharide were presented. However, these neutral sugars occurred in small amounts only (Table 2).

The HPLC molecular mass analysis of F1, F2, and F3 showed broad and incomplete separated peaks. These unresolved molecular mass distribution patterns are typical profiles for polysaccharide conjugates that comprise mixtures of similar building fragments [17]. Besides, the three active anticoagulant fractions were the polydispersity fractions, with the molecular masses between 82 and 281 kDa (Table 3).

F1 was the first fraction purified from the SPE extraction, which had been eluted with 95% water and 5% acetonitrile. The highest yield was obtained for F1, with 54.17% (w/w) of dry crude extract. From the HPLC molecular mass determination analysis, three major not well-separated peaks were identified. The molecular masses were ~280.08 ± 3.57 kDa, ~168.60 ± 3.57 kDa, and ~82.05 ± 3.22 kDa. However, the distinct peak at a retention time of 12.35 min could not be identified by using the set of dextran standards (data not shown). Molecular masses of sample analytes were separated based on the size. Larger molecular mass of analyte was eluted out first, followed by analyte with smaller molecular mass. In addition, retention time for 5 kDa of dextran standard was 10.94 min, while retention time for 27 kDa was 8.20 min. Therefore, this distinct peak with retention time at 12.35 min was postulated to have a molecular mass less than 5 kDa. Besides, fractionation through high water compositions of solvent resulted in the highest amounts of carbohydrates and uronic acids, with 78 ± 2.29% and 31.71 ± 3.96%, respectively. In spite of these, the phenolic acids content was the least abundant in F2 and F3. The percentage for phenolic acids present in F1 was 4.50 ± 0.49%. Meanwhile, F1 contained 2.39 ± 0.11% of rhamnose and 2.39 ± 0.15% of glucose. These results indicated the presence of rhamnose-hexose type polysaccharide.

F2 was fractionated with 90% of water and 10% of acetonitrile containing 14.97% of the total mass. The molecular mass determination analysis for F2 revealed that F2 contains higher molecular mass components than F1. The average molecular mass of two dominant peaks was about ~267.48 ± 0.66 kDa and ~177.36 ± 1.63 kDa. Another distinguishable shoulder tailing peak was about ~224.85 ± 1.28 kDa. The presence of carbohydrates, phenolic acids, and uronic acids pointed out that F2 was a polyphenolic glycoconjugate. There were 46.34 ± 3.86% of carbohydrates, 34.76 ± 0.53% of phenolic acids, and 22.85 ± 3.60% of uronic acids found in F2. The neutral sugars compositions for F2 contained rhamnogalacturonan, homogalacturonan, and rhamnose-hexose types of pectic polysaccharides. The most dominant neutral sugar presence in F2 was rhamnose which was 6.01 ± 0.27%.

F3 was purified with higher compositions of nonpolar solvent, with 80% of water and 20% of acetonitrile, and yielded 8.8% of the total dry weight of the crude extract. The HPLC molecular mass analyses for both F2 and F3 were alike. F3 also contained two dominants and one shoulder tailing peaks with ~271.24 ± 0.39 kDa, ~240.23 ± 0.04 kDa, and 183.49 ± 0.31 kDa, respectively. Phenolic acids were most likely distributed and extracted by higher compositions of nonpolar solvent. Therefore, F3 was rich in phenolic acids compared to F1 and F2. There was 65.12 ± 0.78% of phenolic acids found in F3. On the other hand, the carbohydrates and uronic acids compositions were the least abundant in F3, with 14.98 ± 1.96% and 15.42 ± 2.37%, respectively. Besides, there was only one neutral sugar-galactose found in F3. The percentage of galactose for F3 was 7.05 ± 0.09%, and type of pectic polysaccharide was suggested as homogalacturonan.

**Figure 3:**
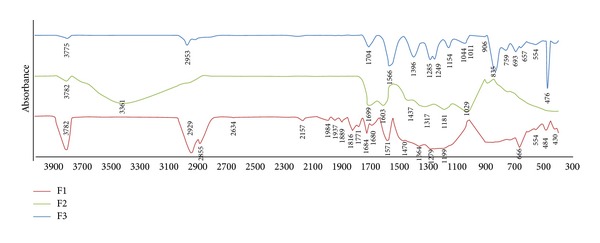
FT-IR spectrum of F1, F2, and F3. Active anticoagulant fractions F1, F2, and F3 were purified by solid phase extraction (SPE) from* Melastoma malabathricum *Linn. leaf hot water crude extract, and fractions were determined to have significantly prolonged activated partial thromboplastin time (APTT). Red line represented F1. Green line represented F2. Blue line represented F3.

The FT-IR spectrum of each active anticoagulant fraction (Figure 3) demonstrated a characteristic carbohydrate group with the signal band at region 3782–3361 cm^−1^. This spectral shape was identified as a stretching vibration of intramolecular hydrogen on hydroxyl (OH) group in carbohydrate [20, 21]. Besides, the band spectra of active anticoagulant fractions (F1, F2, and F3) were found at 2953–2855 cm^−1^. These regions corresponded to the stretch vibration of aliphatic C–H groups derived from the methyl (CH_3_) group [8, 22]. In addition, the characteristic bands of uronic acids or phenolic acids region-carbonyl stretching mode were observed for all three active anticoagulant fractions [23] at 1771–1566 cm^−1^. Stretching of phenolics C–C for F1, F2, and F3 was observed at the bands at 1470–1437 cm^−1^. In addition, the spectra band at 1396–1249 cm^−1^ indicated the presence of phenolic acids in the fractions. These bands were signature bands for phenolics C–O (H) stretching [24].

The FT-IR spectrum of F1 further proved that F1 did contain high amounts of carbohydrates and uronic acids. As shown in Figure 3, F1 spectrum displayed three discrete and intense bands at 3782 cm^−1^, 2929 cm^−1^, and 2855 cm^−1^. The band spectrum at 3782 cm^−1^ was a signature band for carbohydrates. Carbohydrates were also abundantly found at strong signal bands of 1279–1199 cm^−1^. These bands could be due to the ring vibration of carbohydrates overlapping with stretching vibration of OH group and glycosidic vibration [25]. The bands at 2929 cm^−1^ and 2855 cm^−1^ showed the stretching vibration of aliphatic carbon and hydrogen (C–H). The intensive band at 1470 cm^−1^ presented the characteristic of carboxylic functional group for uronic acids or phenolic acids.

The characteristic of carbohydrates, phenolic acids, and uronic acids was displayed at F2 FT-IR spectrum. Broad band signal was exhibited at region 3361 cm^−1^. This band corresponded to the interaction of hydroxyl (O–H) group of carbohydrates and phenolic acids. Meanwhile, the intensive signals at 1699–1603 cm^−1^ demonstrated the stretching vibration of carboxylic group of uronic acids or phenolic acids in asymmetric and symmetric modes. Typical galacturonopyranose backbone of pectic polysaccharides was also presented at region 1181 cm^−1^.

F3, with the least abundance of carbohydrate moiety, did not have intense signal bands at the carbohydrate regions. Nevertheless, F3 did display a distinct band at 1566 cm^−1^. This region matched the characteristic of uronic acids or phenolic acids. Signals at region 1285–1249 cm^−1^ matched the FT-IR spectrum for phenolic acids. Moreover, spectra bands at 1044–1011 cm^−1^ displayed the stretching vibration of C–O for phenolic acids [26].

**Figure 4:**
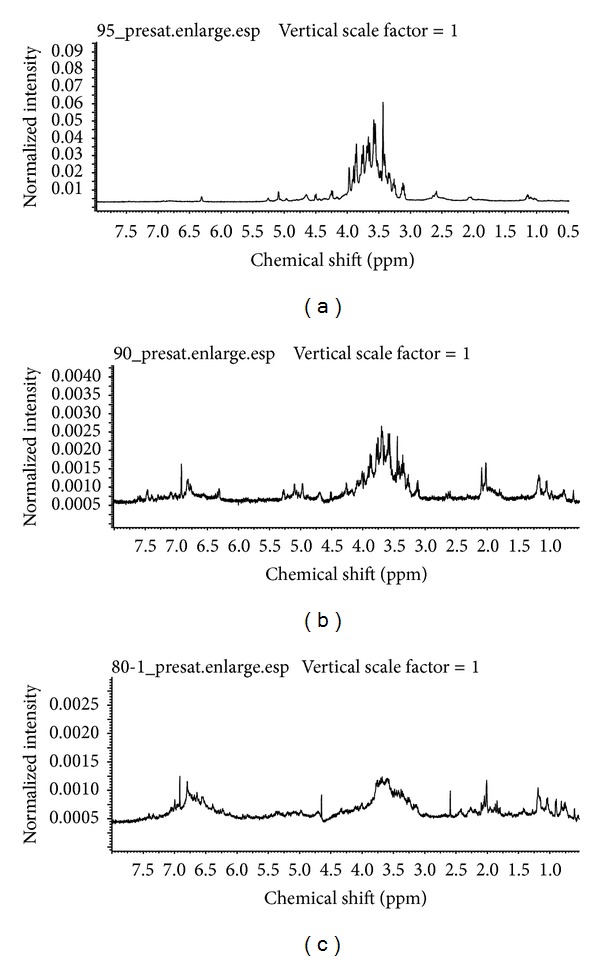
^1^H NMR spectrum of F1 (a), F2 (b), and F3 (c). Active anticoagulant fractions F1, F2, and F3 were purified by solid phase extraction (SPE) from* Melastoma malabathricum *Linn. leaf hot water crude extract and were determined with significantly prolonged activated partial thromboplastin time (APTT).

In agreement with the general chemical characteristics and FT-IR spectra, the ^1^H NMR spectra for active anticoagulant fractions (F1, F2, and F3) also revealed the presence of carbohydrates and phenolic acids. Overall, the ^1^H NMR spectra were shown to be complex and displayed broad hump signals. These were due to the complex nature of F1, F2, and F3. The not well-resolved spectra were also caused by the overlapping of many chemical constituents within the samples. From Figure 4, carbohydrate moieties were observed for F1, F2, and F3, and the carbohydrate regions were observed at *δ* 5.30–3.0 ppm.

The ^1^H NMR spectrum for F1 was slightly different from that of ^1^H NMR of F2 and F3. F1, which was rich in carbohydrate contents, only displayed a complex signal at *δ* 5.30–3.0 ppm. This suggests the presence of C2–C6 atoms attached at the carbohydrate pyranose ring [18]. Other than that, both F2 and F3 showed the existence of phenolic acids within the fractions at *δ* 8.0–6.5 ppm. Both consisted of higher phenolic acids contents compared to F1. Furthermore, with the highest amount of phenolic acids, F3 was translated into having a more complicated signal than F2. The additional ^1^H NMR signals at *δ* 2.1–1.9 observed for F2 and F3 could be due to the substitution of acetyl, ethyl, or methyl groups for carbohydrates [27].

Generally, F1, F2, and F3 showed significant prolonged blood clotting time, especially in the intrinsic pathway. The anticoagulant activities of the fractions were in an increasing manner of F1 > F2 > F3. These fractions were purer and they gave better anticoagulant activity profile compared to the crude extract. Taken altogether, and with all the chemical analyses, the results showed that active anticoagulant* M. malabathricum *Linn. fractions had high polydispersity of macromolecules. The active principles of the anticoagulant activity of these fractions were acidic polysaccharides and polyphenolics. This result further proved that the anticoagulant activities for* M. malabathricum *Linn. fractions were high when the polysaccharide and hexuronic acids contents were high. A plausible explanation for the anticoagulant activities is the presence of carboxylic acid from the uronic acids, which provided negative charge properties to the fractions [18]. Hence, the results of this study are in agreement with several reported works, whereby higher plants such as* Lythrum salicaria, Erigeron canadensis, *and* Porana volubilis *have been found to contain polyphenolic polysaccharide conjugates as negative charged macromolecules for anticoagulant activity [9, 18]. Even though the hexuronic acid and polysaccharides contents were moderate for F3, its anticoagulant activity is still promising. Mozzicafreddo et al. [19], Guglielmone et al. [28], and Dong et al. [29] reported that polyphenolic demonstrated anticoagulant activity through prolongation of activated partial thromboplastin time (APTT). In this regard, we postulate that probably the anticoagulant activity of F3 could be due to its high polyphenolic contents.

## 4. Conclusion

All in all, the presence of negative charged polyphenolic-polysaccharides were suggested to have played a role in the anticoagulant activity, especially prolonging blood coagulation in the intrinsic pathway, as exhibited by the purified* M. malabathricum* Linn. hot water leaf extract. This discovery opens up the avenue that the simple preparation of fractions and the abundant availability of* M. malabathricum* Linn. plant materials could lead to the development of* M. malabathricum *Linn. active anticoagulant fractions as safe and cheap natural oral anticoagulant agents. A further study to isolate single anticoagulant bioactive compound from active anticoagulant SPE purified fractions (F1, F2, and F3) and an investigation into the action of single bioactive compound in blood coagulation pathway can be carried out.

## Figures and Tables

**Table 1:** Chemical compositions of active anticoagulant fractions of F1, F2, and F3 were purified through solid phase extraction (SPE) from *Melastoma malabathricum *Linn. leaf hot water extract. Carbohydrate content was measured by using phenol-sulphuric acid, while glucose was used as a standard. Phenolic acid was measured by using the Folin-Ciocalteu method, and gallic acid was used as a standard. Uronic acid was measured by carbazole assay, and glucuronic acid was used as a standard. Values are expressed as mean ± S.E.M. (*n* = 3).

	Total yield (%)	Carbohydrate content (wt%)	Phenolic acid content (wt%)	Uronic acid content (wt%)
F1	54.17	78 ± 2.29	4.59 ± 0.49	31.71 ± 3.96
F2	14.97	46.34 ± 3.86	34.76 ± 0.53	22.85 ± 3.60
F3	8.8	14.98 ± 1.96	65.12 ± 0.78	15.42 ± 2.37

**Table 2:** Neutral sugar compositions of active anticoagulant fractions of F1, F2, and F3 were purified through solid phase extraction (SPE) from the hot water extract of *Melastoma malabathricum *Linn. leaf. Neutral sugar compositions of the samples were determined by using the GC-FID analysis. Values are expressed as mean ± S.E.M. (*n* = 3).

	Neutral sugar compositions		
	Glu (wt%)	Gal (wt%)	Rhamn (wt%)
F1	2.39 ± 0.15	n.d.	2.39 ± 0.11
F2	1.05 ± 0.10	1.83 ± 0.22	6.01 ± 0.27
F3	n.d.	7.05 ± 0.09	n.d.

Glucose is labelled as Glu; galactose is labelled as Gal; rhmanose is labelled as Rhamn; not detected is labelled as n.d.

**Table 3:** Molecular mass of active anticoagulant fractions of F1, F2, and F3 was purified through solid phase extraction (SPE) from *Melastoma malabathricum *Linn. leaf hot water extract. Molecular mass was determined by gel permeable chromatography. There were three distinguishable peaks identified from the gel permeable chromatogram of each fraction and which was labelled as Peak 1, Peak 2, and Peak 3. A set of dextran standards was used for molecular mass determination of the three peaks. Values are expressed as mean ± S.E.M. (*n* = 3).

	Molecular mass		
	10^3 ^(kDa)		
	Peak 1	Peak 2	Peak 3
F1	280.08 ± 3.57	168.60 ± 3.57	82.05 ± 3.22
F2	267.48 ± 0.66	224.85 ± 1.28	177.36 ± 1.63
F3	271.24 ± 0.39	240.23 ± 0.04	183.49 ± 0.31
